# The Western Lancet, and the Memphis School Advertisement

**Published:** 1850-10

**Authors:** 


THE WESTERN LANCET, AND THE MEMPHIS SCHOOL ADVERTISEMENT.

Our friends of the Western Lancet express some concern respecting the advertisement of the Memphis School, on the cover of this Journal. There should be some discrimination in these censures, and a little examination would have taught the Lancet, that the editors of the Western Journal are not the owners of the work. The advertising is the property of the proprietors of the Journal, and is a matter upon which the editors are not consulted. It will be time enough to censure the editors, when the Memphis School figures in the contents of the Journal. B.

